# Transitional probabilities outweigh frequency of occurrence in statistical learning of simultaneously presented visual shapes

**DOI:** 10.3758/s13421-024-01665-x

**Published:** 2024-12-10

**Authors:** Ansgar D. Endress

**Affiliations:** Department of Psychology, City St George’s, University of London, Northampton Square, London, EC1V 0HB UK

**Keywords:** Statistical learning, Declarative memory, Language acquisition, Hebbian learning

## Abstract

Statistical learning is a mechanism for detecting associations among co-occurring elements in many domains and species. A key controversy is whether it leads to memory for discrete chunks composed of these associated elements, or merely to pairwise associations among elements. Critical evidence for the mere-association view comes from the “phantom-word” phenomenon, where learners recognize statistically coherent but unattested items better than actually presented items with weaker internal associations, suggesting that they prioritize pairwise associations over memories for discrete units. However, this phenomenon has only been demonstrated for sequentially presented stimuli, but not for simultaneously presented visual shapes, where learners might prioritize discrete units over pairwise associations. Here, I ask whether the phantom-word phenomenon can be observed with simultaneously presented visual shapes. Learners were familiarized with scenes combining two triplets of visual shapes (hereafter “words”). They were then tested on their recognition of these words vs. part-words (attested items with weaker internal associations), of phantom-words (unattested items with strong internal associations) vs. part-words, and of words vs. phantom-words. Learners preferred both words and phantom-words over part-words and showed no preference for words over phantom-words. This suggests that, as for sequentially input, statistical learning in simultaneously presented shapes leads primarily to pairwise associations rather than to memories for discrete chunks. However, as, in some analyses, the preference for words over part-words was slightly higher than for phantom-words over part-words, the results do not rule out that, for simultaneous presented items, learners might have some limited sensitivity to frequency of occurrence.

**Supplementary Information:**

The online version contains supplementary material available at 10.3758/s13421-024-01665-x.

## Introduction

Statistical learning is a ubiquitous learning mechanism, enabling learners to detect, and possibly utilize, co-occurrence relations among elements. For example, when exposed to a continuous stream of syllables, learners might group syllables that frequently co-occur into units that correspond to words (Aslin, Saffran, & Newport, 1998; Saffran, Aslin, & Newport, 1996a). Likewise, in vision, learners might associate shapes that often co-occur within scenes, which might facilitate the recognition of objects composed of those shapes (e.g., Fiser & Aslin, 2005; Orbán, Fiser, Aslin, & Lengyel, 2008).

The initial motivation of statistical learning was that it provided one of the few plausible bottom-up mechanisms for extracting and memorizing recurring units like words from continuous sequences (e.g., Erickson, Thiessen, & Estes, 2014; Graf-Estes, Evans, Alibali, & Saffran, 2007; Hay, Pelucchi, Graf Estes, & Saffran, 2011; Isbilen, McCauley, Kidd, & Christiansen, 2020; Karaman & Hay, 2018; Perruchet, 2019; Shoaib, Wang, Hay, & Lany, 2018). However, the question of whether statistical learning truly facilitates the memorization of these units is controversial. An alternative view proposes that statistical learning primarily supports the formation of pairwise associations among co-occurring elements (e.g., syllables) rather than the memorization of units (Endress & de Seyssel, under review; Endress, Slone, & Johnson, 2020).

A key piece of evidence for the mere-associations view is the phantom-word phenomenon. In this paradigm, learners can recognize spurious “units” that have not been presented during a sequence, but that have the same statistical properties as units that have been presented (e.g., Endress & Langus, 2017; Endress & Mehler, 2009, but see Perruchet & Poulin-Charronnat, 2012). If learners are more familiar with these spurious phantom-words than with items that actually occurred in the speech stream (but have weaker internal associations), such results seem to suggest that learners just tracked the associations among elements, but did not memorize any units. After all, they preferred unattested items over attested items if the former had stronger internal associations – even though unattested items are unlikely to have memory representations.

However, while this phenomenon has been observed for *sequentially* presented items in both vision and audition, there is evidence that for simultaneously presented visual shapes, units might actually be memorized. This raises the question of whether the phantom-word phenomenon can be replicated with simultaneously presented items. If statistical learning of simultaneously presented visual shapes leads only to the recognition of pairwise associations without memorizing the units as wholes, such results would strongly support the mere-associations view.

### Memory vs. mere associations in sequential learning

In statistical learning tasks, participants are typically exposed to statistically structured sequences of stimuli, such as syllables, shapes, or other elements. These sequences contain statistical regularities, such as transitional probabilities (TPs) between elements, which participants can use to identify recurring patterns. TPs represent the conditional probability of an element $\sigma _2$ occurring after another element $\sigma _1$ within a sequence. Mathematically, this can be represented as $\text {TP}(\sigma _2|\sigma _1) = \frac{{\text {Count}(\sigma _1\sigma _2)}}{{\text {Count}(\sigma _1)}}$, where $\text {Count}(\sigma _1\sigma _2)$ represents the number of times the sequence $\sigma _1\sigma _2$ occurs, and $\text {Count}(\sigma _1)$ represents the number of times the element $\sigma _1$ occurs.

Following exposure to such sequences, the participants’ ability to detect the statistical structures is tested in a recognition test contrasting items with stronger TPs and items with weaker TPs. For example, in Saffran and colleagues’ (Aslin et al., 1998; Saffran, Newport, & Aslin, 1996b; Saffran, Aslin, & Newport, 1996a) seminal experiments, participants were presented with a continuous stream of syllables without explicit word boundaries. Unbeknownst to the participants, the stream contained statistically defined “words” with strong word-internal TPs. After exposure to such streams, participants were tested on their ability to discriminate between high- and low-probability syllable sequences (using techniques appropriate for the infant or adult participants). The participants’ ability to choose high-TP items over low-TP items demonstrates their sensitivity to the statistical regularities present in the stream.

While a sensitivity to statistical structure has been widely observed across various modalities, including speech, audition, vision, and touch, as well as in non-human animals (e.g., Aslin et al., 1998; Batterink & Paller, 2017; Bulf, Johnson, & Valenza, 2011; Chen & Ten Cate, 2015; Conway & Christiansen, 2005; Creel, Newport, & Aslin, 2004; Endress, 2010; Endress & Wood, 2011; Fiser & Aslin, 2002, 2005; Fló, Benjamin, Palu, & Dehaene-Lambertz, 2022; Glicksohn & Cohen, 2011; Hauser, Newport, & Aslin, 2001; Kirkham, Slemmer, & Johnson, 2002; Saffran, Newport, & Aslin, 1996b; Saffran, Aslin, & Newport, 1996a; Saffran, Johnson, Aslin, & Newport, 1999; Saffran & Griepentrog, 2001; Sohail & Johnson, 2016; L.K. Slone & Johnson, 2015; L.K. Slone & Johnson, 2018; Tompson, Kahn, Falk, Vettel, & Bassett, 2019; Toro, Trobalon, & Sebastián-Gallés, 2005; Turk-Browne, Jungé, & Scholl, 2005; Turk-Browne & Scholl, 2009), the interpretation of such results remains contentious, particularly regarding whether this process leads to the memorization of entire units or merely the formation of pairwise associations among elements. Given the focus of the current paper, I will focus more on this mere-associations view. For a critical discussion of the evidence supporting the memory view as well as alternative interpretations thereof, see Endress and de Seyssel (under review) and Endress et al. (2020).

Support for the mere-association view comes from several key observations, including computational modeling of behavioral and electrophysiological statistical learning results with memory-less Hebbian mechanisms (Endress & Johnson, 2021; Endress, 2024), and an almost complete inability to consciously recall statistical defined items such as words even when their statistical structure has been demonstrably learned (Batterink, 2020; Endress & de Seyssel, under review).

Most relevant to the current experiment, participants can recognize unattested items that did not occur during the familiarization sequences, and can prefer them over items that did occur during familiarization. Such items include items played backwards with respect to the familiarization sequence (e.g., Endress & Wood, 2011; Turk-Browne & Scholl, 2009; Jones & Pashler, 2007), as well as “phantom-words” (see below; Endress & Langus, 2017; Endress & Mehler, 2009). This ability to recognize items that were not presented during familiarization but have similar statistical properties as those items that were presented suggests that a recognition test is not necessarily diagnostic of memory processes. This, in turn, supports the notion that participants might just form associations between elements rather than memorizing entire units. After all, one cannot form memories of items that have not been encountered (though it is possible to implant false memories of course, see e.g., Loftus & Pickrell, 1995, and, as I will argue in the discussion, recognizing unattested items is critical for generalization).

More specifically, in (visual or auditory) phantom-word experiments, participants were presented with sequences of stimuli designed to contain statistically defined “words” as well as spurious “phantom-words” that had identical statistical properties as the words but were not actually presented during the sequence (Endress & Langus, 2017; Endress & Mehler, 2009). Participants preferred such phantom-words to lower-probability items that did actually occur in the familiarization sequences (Endress & Langus, 2017; Endress & Mehler, 2009), and, at least in some experiments, were unable to discriminate between phantom-words and items with identical TPs that were presented during the familiarization sequence (Endress & Langus, 2017; Endress & Mehler, 2009, but see Perruchet & Poulin-Charronnat, 2012). Again, if participants prefer unattested high-TP items over low-TP items they have actually encountered, such preferences in a recognition test cannot be diagnostic of the memorization of statistically defined units.

### Memory vs. mere associations in simultaneous displays

While the evidence for memory processes in statistical learning tasks from *sequential* input remains contentious, there is more compelling support for the view that statistical learning might lead to memories of entire units in the case of simultaneous visual displays. For example, in statistical learning tasks, participants often exhibit better recognition of entire units compared to sub-units. For example, if the elements *ABC* form a statistically defined unit, participants sometimes find it easier to recognize the entire *ABC* unit compared to its sub-units *AB* or *BC* (e.g., Fiser & Aslin, 2005; Giroux & Rey, 2009; Orbán et al., 2008; Slone & Johnson, 2018).

However, both Fiser and Aslin (2005) and Slone and Johnson (2018) found such results only in some of their experiments, and not others. Further, better recognition of units than of sub-units can be reproduced by memory-less Hebbian models (Endress and de Seyssel, under review), and attentional processes may also contribute to the preference for units over sub-units (Endress, in preparation). Such results suggest that these preferences might be less diagnostic of memory processes than initially thought. Given these discrepant explanations for the observed effects, it is important to provide another critical test of the view that statistical learning leads to memory for statistically defined items. I thus ask whether the phantom-word phenomenon can also be observed in studies involving simultaneous visual displays.

### The current experiment

In the current experiment, I seek to replicate the phantom-word phenomenon with simultaneously presented visual shapes.

Participants were familiarized with visual scenes combining two (statistically defined) “words” of three shapes each. (I refer to shape combinations as words for consistency with the earlier literature.) The scenes were designed as to allow for the creation of “phantom-words.“

Following this familiarization, participants were tested on three types of test-trials. First, they had to choose between words and “part-words.” Part-words are shape combinations that appeared during familiarization, but whose shapes came from different words, and thus had weaker TPs than actual words. In line with much of the statistical learning literature, I used this contrast to establish a sensitivity to statistical structure.

Second, participants had to choose between phantom-words and part-words. I expected to replicate a preference for phantom-words over part-words, showing that participants weigh TPs higher than frequency of occurrence, which in turn would suggest that they do not encode words in memory.

Third, participants had to choose between words and phantom-words. While participants showed no preference for words over phantom-words in some earlier experiments (see above), one would expect a strong preference for actual words if participants truly memorized them in the case of simultaneously presented visual items.

**Fig. 1:**
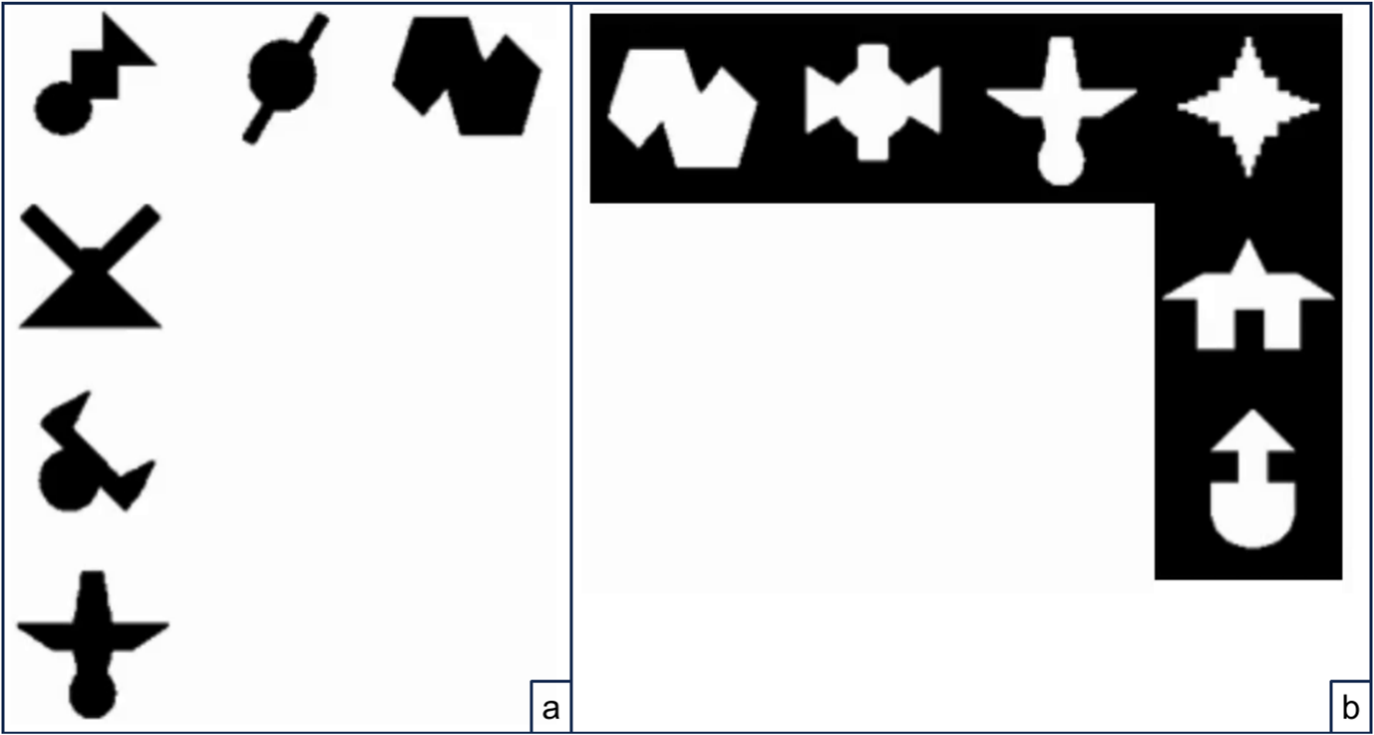
Example configurations presented during familiarizations, with **a** black shapes on a white background and **b** white shapes on a black background. Presenting black shapes on a white background is the standard presentation mode in statistical learning tasks. Presenting white shapes on a black background was intended to make the shapes appear as part of a whole. However, this manipulation was unsuccessful

I also applied an orthogonal manipulation that turned out not to affect the results. Specifically, for half of the participants, the shapes were presented as black figures on a white background. This is the usual mode of presentation in the statistical learning literature, and might encourage the perception of the scenes as a collection of separate shapes (see Fig. 1a). For the other participants, shapes were presented as white “holes” on a black background (see Fig. 1b). The motivation was to encourage participants to perceive the shapes as holes in a single object (i.e., the black background), which, in turn, might encourage memorization of these wholes, and thus of entire units. However, this manipulation was unsuccessful, maybe because the polarity inversion did not provide convincing 3D cues. I thus include the polarity type in the analyses below, but do not discuss it further.

**Table 1:** Demographics of the full sample and the restricted sample, where those participants were excluded whose accuracy on word vs. part-words trials did not exceed 50%

Population	Sample type	Color polarity	*N*	Females	Males	Age	Age range
Main (testable)	Full sample	black on white	82	47	35	30.3	19-66
Main (testable)	Full sample	white on black	79	33	46	30.5	18-57
Pilot (students)	Full sample	black on white	23				
Pilot (students)	Full sample	white on black	27				
Main (testable)	Restricted sample	black on white	57	32	25	30.7	19-59
Main (testable)	Restricted sample	white on black	51	22	29	31.9	18-57
Pilot (students)	Restricted sample	black on white	12				
Pilot (students)	Restricted sample	white on black	15				

For the student sample, age and gender have not been recorded due to experimenter error

## Materials and methods

### Participants

The main experiment recruited participants from testable minds (https://minds.testable.org/). A pilot experiment recruited participants from first-year students at City St George’s, University of London (UK). In the latter population, other experiments where attention checks can be implemented typically need to exclude a substantial proportion of the sample due to insufficient attention. Unfortunately, the present experiment does not offer a clear performance-based criterion to make sure that participants paid attention to the stimuli, as the task might be genuinely difficult. However, given that my main interest lies in the performance on trials involving phantom-words for participants who succeeded in the statistical learning task, it is more conservative to exclude participants who might not have paid attention to the task, even if this leads to an overestimation of their statistical learning abilities.

As a result, I rely on the assumption that earlier statistical learning literature has shown that participants can learn statistical relations *in principle*, and exclude those participants not exceeding an accuracy of 50% on word vs. part-word trials. This criterion led to the removal of 53 and 23 participants from the testable minds and students samples, respectively. I present the results from these restricted samples jointly with the results from the full sample. The pattern of significance was very similar when all participants were included (see below). The demographics of the full samples as well as the restricted samples are given in Table 1. In the student sample, age and gender were not recorded due to experimenter error. Results from the student sample are reported in Supplementary Online Material SM2.

As I had no *a priori* estimates of the expected effect sizes (and as the resulting sample size calculations can be problematic; Pek, Pitt, & Wegener, 2024), sample sizes were determined by the available funding (for the main sample) and by the number of available students (for the pilot sample). However, a sensitivity analysis for the main sample indicates that the sample sizes – 161 in the full sample and 108 in the restricted sample – are sufficient to detect effect sizes (Cohen’s *d*) of 0.20 and 0.25, respectively, with 80% power. These detectable effect sizes are much smaller than the critical effect sizes reported below, suggesting that the sample sizes were sufficient to detect the critical effects.

**Table 2:** Design of the actual words and phantom-words

**Phantom-word: ABC**		
ABI	GBC	AHC
**Phantom-word: DEF**		
DEI	GEF	DHF
**Phantom-word: JKL**		
JKR	PKL	JQL
**Phantom-word: MNO**		
MNR	PNO	MQO

Actual words were generated from phantom-words by replacing one of their shapes

**Fig. 2:**
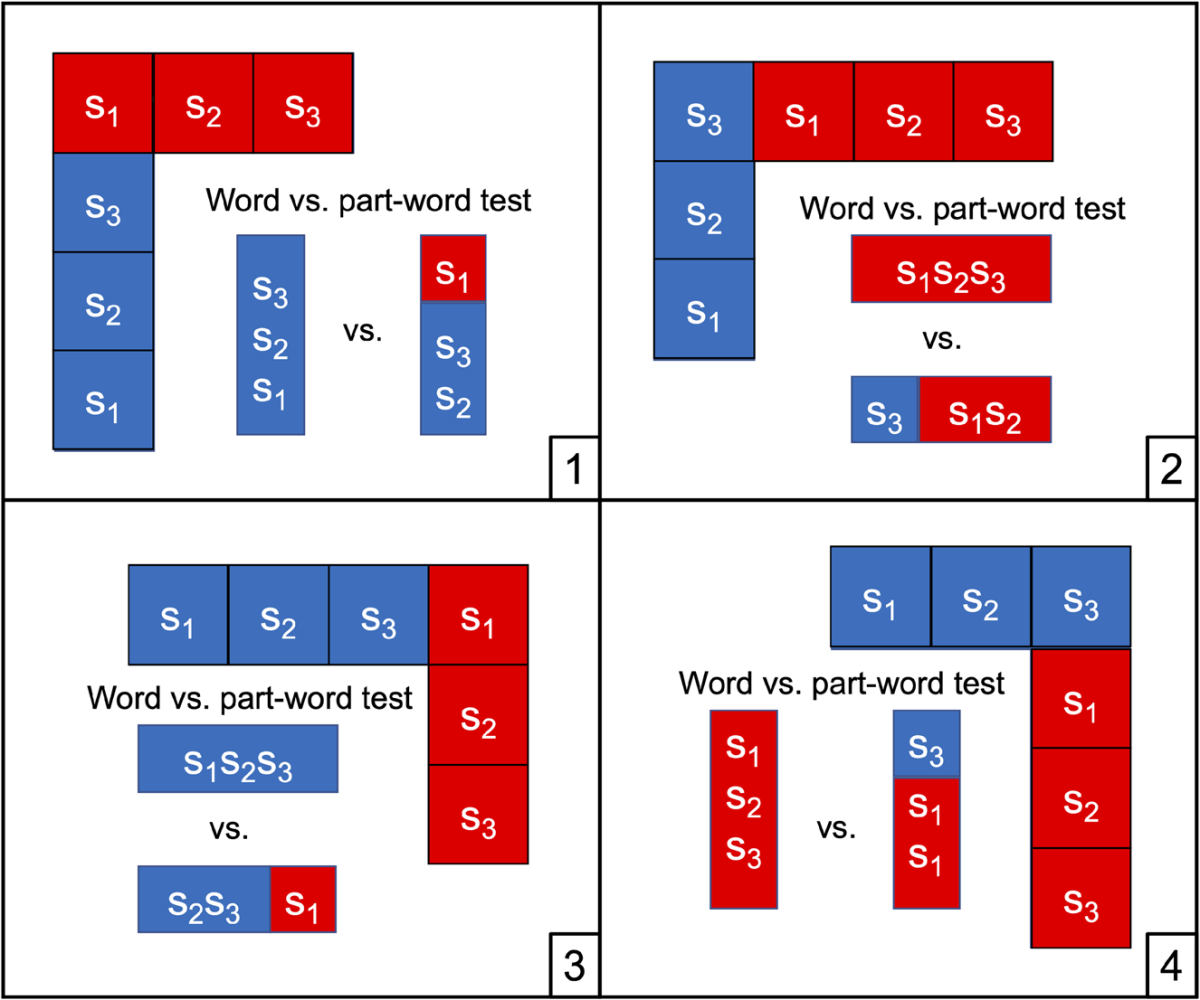
Configurations used in the familiarization scenes. Each box represents a shape. Shapes belonging to the same word are colored in the same color. All scenes were composed of one horizontally and one vertically arranged “word.” During the test phase, one type of part-word was extracted from each configuration. For example, in Configuration 1 (*top left*), the part-word consisted of the left-most shape of the horizontally arranged word and the two top-most shapes of the vertically arranged word

### Apparatus

The experiment was run on testable.org.

### Materials

The stimuli were the visual shapes used by Fiser and Aslin (2002). I used a total of 18 shapes to generate 12 units (and phantom-units). These shapes were randomly chosen from a total set of 24 shapes (see below). Individual shapes appeared as *bmp* images with a size of 74 $\times$ 74 pixels. However, the actual size of the shapes on the participants’ displays is unknown due to online administration of the experiment.

Locations of shapes within scenes were pre-calculated offline. Scenes were composed online by testable.

### Design and familiarization

#### Creating words from phantom-words

As shown in Table 2, phantom-words were generated following the design in Endress and Mehler (2009) and Endress and Langus (2017). Specifically, I reserved 12 shapes to generate two sets of two phantom-words each. Within each set of phantom-words, I reserved another set of three shapes to generate the actual words, by replacing one shape at a time. For example, and as shown in Table 2, if *ABC* and *DEF* are two *phantom-words* (where each letter represents a shape), the corresponding *actual words* would be *GBC* and *GEF* (replacing the first element of the phantom-words), *AHC* and *DHF* (replacing the middle element of the phantom-words), as well as *ABI* and *DEI* (replacing the last element of the phantom-words). I generated ten different random assignments between shapes and units, corresponding to different “languages” in statistical learning tasks.

#### Combining words into scenes

Familiarization scenes were created according to the four configurations shown in Fig. 2. Each scene comprised two words. These words came from different sets, where a “set” of words refers to those words that can be generated from the same phantom-word by substitution of a single shape. As shown in Fig. 2, one word in each scene was presented vertically, while a second word was presented horizontally above the first. The scenes differed in whether the words were stacked on top of each other or placed next to each other, and in whether the bottom word appeared on the left or right.

As I used all combinations of the six words in each set, with each of the four configurations, and with each word appearing on each of the two (left or right) sides of the configurations in Fig. 2, one obtains $6 \times 6 \times 4 \times 2 = 288$ scenes. As a result, each word appeared 48 times in total, and 24 times in each (horizontal or vertical) orientation. Similarly, all shapes occurred equally often during familiarization.

Before starting the familiarization, participants were informed that the study aimed to investigate how individuals remember combinations of objects. They were told that they would be shown a series of scenes displaying combinations of objects and instructed to pay attention to these scenes. Following this, each scene was presented once for 2000 ms and with an ITI of 1000 ms, leading to a familiarization duration of 14 min 24 s.

### Test

As mentioned above, learning was assessed during two-alternative forced-choice tests. Participants were informed that they would be presented with pairs of new scenes containing fewer objects. They were told that, in each pair, one scene was embedded in the scenes they had viewed previously, while the other was not. They were asked to indicate which scenes looked more familiar, by clicking on one of two buttons corresponding to the first or the second scene, respectively.

Following this, all participants then completed three types of test trials in a single intermixed block: Choices between words and part-words, between phantom-words and part-words, and between words and phantom-words.

Test items were presented at the center of the screen rather than in their original positions and were shown one after the other, for a total of 36 trials. I will now describe the different test types.

#### Words vs. part-words

As shown in Fig. 2, each configuration allows for exactly one part-word, by combining adjacent shapes from the two underlying words. For example, in Configuration 1, the only part-word without a bend uses the two top-most shapes from the vertical word and the left-most shapes from the horizontal word.

I randomly selected 12 combinations of words to create the test trials. One word in each combination came from either set. (As mentioned above, a “set” of words refers to those words that can be generated from the same phantom-word by substitution of a single shape.) Each word appeared equally often either as the left or as the right item in the configurations in Fig. 2. I randomly paired these word combinations with a configuration and generated the corresponding words and part-words. Each configuration was used equally often. As a result, each word occurred twice, and each part-word once.

The order was randomly chosen; an equal number of trials started with words and part-words, respectively. Participants completed 12 of these test trials in total.

Compared to the horizontal orientation, vertical shape combinations were rotated by 90 degrees to the left when the vertical shape combinations appeared on the left (i.e., in Configurations 1 and 2), and by 90 degrees to the right when the vertical shape combinations appeared on the right (i.e., in Configurations 3 and 4). The shapes were not rotated.

#### Phantom-words vs. part-words

For the phantom-word vs. part-word test, I reused the same trials as in the word vs. part-word test, except that words were replaced with the corresponding phantom-words. As a result, each phantom-word occurred three times, with each part-word occurring only once, for a total of 12 trials.

#### Words vs. phantom-words

In the word vs phantom-word test, I presented all words and their corresponding phantom-words. As a result, each word occurred once, and each phantom-word three times. Orientations were chosen randomly. This yields a total of 12 trials.

### Analysis

I analyzed the results in two ways. First, I compared the performance in the different trial types to the chance level of 50% using a Wilcoxon test. To compare performance across trial types, I calculated normalized difference scores, that is, $\frac{\text {accuracy}_{\text {trial type 1}} - \text {accuracy}_{\text {trial type 2}}}{\text {accuracy}_{\text {trial type 1}} + \text {accuracy}_{\text {trial type 2}}}$, indicating whether performance in one trial type is better than in the other. These difference scores were then compared to the chance level of zero, again using Wilcoxon tests. I also asked whether any of these results were affected by the color polarity type (i.e., black on white vs. white on black). Following Rosenthal, Rosnow, and Rubin (1999), I used these focused analyses to target the contrasts of interest, ensuring that the visualizations matched the statistical tests.

Second, I confirmed these results using a set of generalized linear mixed models with the fixed factor predictors trial type and color polarity as well as their interaction, and a random intercept for participants. I fitted separate model for each (full vs. restricted) sample and trial contrast (word vs. part-word trials vs. word vs. phantom-word trials and word vs. part-words and phantom-word vs. part-word trials).

Results from the much larger main sample will be presented in the main text. Results from the student sample will be presented in Supplementary Online Material SM2.

**Table 3:** Descriptives of accuracy scores and difference scores for the main sample

Trial type	*M*	*SE*	$p_{\text {Wilcoxon}}$	$p_{\text {HB}}$	*r*
**Full sample - color polarities combined (N = 161)**					
Words vs. Part-Words	60.093	1.281	< 0.001	< 0.001	0.550
Words vs. Phantom-Words	48.292	1.158	0.571	1.000	0.045
Phantom-Words vs. Part-Words	58.333	1.303	< 0.001	< 0.001	0.453
$\frac{\text {Words vs. Part-Words} - \text {Words vs. Phantom-Words}}{\text {Words vs. Part-Words} + \text {Words vs. Phantom-Words}}$	0.111	0.016	< 0.001	< 0.001	0.488
$\frac{\text {Words vs. Part-Words} - \text {Phantom-Words vs. Part-Words}}{\text {Words vs. Part-Words} + \text {Phantom-Words vs. Part-Words}}$	0.016	0.014	0.519	1.000	0.051
**Full sample - black on white (N = 82)**					
Words vs. Part-Words	60.671	1.795	< 0.001	< 0.001	0.550
Words vs. Phantom-Words	47.764	1.633	0.495	1.000	0.075
Phantom-Words vs. Part-Words	56.606	1.738	< 0.001	0.005	0.387
$\frac{\text {Words vs. Part-Words} - \text {Words vs. Phantom-Words}}{\text {Words vs. Part-Words} + \text {Words vs. Phantom-Words}}$	0.120	0.022	< 0.001	< 0.001	0.509
$\frac{\text {Words vs. Part-Words} - \text {Phantom-Words vs. Part-Words}}{\text {Words vs. Part-Words} + \text {Phantom-Words vs. Part-Words}}$	0.034	0.019	0.147	1.000	0.160
**Full sample - white on black (N = 79)**					
Words vs. Part-Words	59.494	1.849	< 0.001	< 0.001	0.548
Words vs. Phantom-Words	48.840	1.660	0.869	1.000	0.019
Phantom-Words vs. Part-Words	60.127	1.951	< 0.001	< 0.001	0.517
$\frac{\text {Words vs. Part-Words} - \text {Words vs. Phantom-Words}}{\text {Words vs. Part-Words} + \text {Words vs. Phantom-Words}}$	0.101	0.023	< 0.001	0.001	0.458
$\frac{\text {Words vs. Part-Words} - \text {Phantom-Words vs. Part-Words}}{\text {Words vs. Part-Words} + \text {Phantom-Words vs. Part-Words}}$	-0.003	0.021	0.701	1.000	0.043
**Full sample - Effect of color polarity**					
Words vs. Part-Words			0.607	1.000	0.041
Words vs. Phantom-Words			0.458	1.000	0.058
Phantom-Words vs. Part-Words			0.227	1.000	0.095
$\frac{\text {Words vs. Part-Words} - \text {Words vs. Phantom-Words}}{\text {Words vs. Part-Words} + \text {Words vs. Phantom-Words}}$			0.519	1.000	0.051
$\frac{\text {Words vs. Part-Words} - \text {Phantom-Words vs. Part-Words}}{\text {Words vs. Part-Words} + \text {Phantom-Words vs. Part-Words}}$			0.263	1.000	0.088
**Restricted sample - color polarities combined (N = 108)**					
Words vs. Part-Words	69.136	1.011	NA	NA	NA
Words vs. Phantom-Words	49.306	1.432	0.903	1.000	0.012
Phantom-Words vs. Part-Words	60.957	1.587	< 0.001	< 0.001	0.556
$\frac{\text {Words vs. Part-Words} - \text {Words vs. Phantom-Words}}{\text {Words vs. Part-Words} + \text {Words vs. Phantom-Words}}$	0.180	0.015	< 0.001	< 0.001	0.776
$\frac{\text {Words vs. Part-Words} - \text {Phantom-Words vs. Part-Words}}{\text {Words vs. Part-Words} + \text {Phantom-Words vs. Part-Words}}$	0.075	0.015	< 0.001	< 0.001	0.419
**Restricted sample - black on white (N = 57)**					
Words vs. Part-Words	69.152	1.395	NA	NA	NA
Words vs. Phantom-Words	49.269	2.065	0.905	1.000	0.016
Phantom-Words vs. Part-Words	58.626	1.985	< 0.001	0.001	0.531
$\frac{\text {Words vs. Part-Words} - \text {Words vs. Phantom-Words}}{\text {Words vs. Part-Words} + \text {Words vs. Phantom-Words}}$	0.182	0.021	< 0.001	< 0.001	0.760
$\frac{\text {Words vs. Part-Words} - \text {Phantom-Words vs. Part-Words}}{\text {Words vs. Part-Words} + \text {Phantom-Words vs. Part-Words}}$	0.091	0.021	< 0.001	0.001	0.518
**Restricted sample - white on black (N = 51)**					
Words vs. Part-Words	69.118	1.496	NA	NA	NA
Words vs. Phantom-Words	49.346	2.012	0.985	1.000	0.003
Phantom-Words vs. Part-Words	63.562	2.516	< 0.001	< 0.001	0.584
$\frac{\text {Words vs. Part-Words} - \text {Words vs. Phantom-Words}}{\text {Words vs. Part-Words} + \text {Words vs. Phantom-Words}}$	0.178	0.021	< 0.001	< 0.001	0.797
$\frac{\text {Words vs. Part-Words} - \text {Phantom-Words vs. Part-Words}}{\text {Words vs. Part-Words} + \text {Phantom-Words vs. Part-Words}}$	0.056	0.021	0.026	0.238	0.311
**Restricted sample - Effect of color polarity**					
Words vs. Part-Words			0.959	1.000	0.005
Words vs. Phantom-Words			0.878	1.000	0.015
Phantom-Words vs. Part-Words			0.125	1.000	0.148
$\frac{\text {Words vs. Part-Words} - \text {Words vs. Phantom-Words}}{\text {Words vs. Part-Words} + \text {Words vs. Phantom-Words}}$			0.784	1.000	0.026
$\frac{\text {Words vs. Part-Words} - \text {Phantom-Words vs. Part-Words}}{\text {Words vs. Part-Words} + \text {Phantom-Words vs. Part-Words}}$			0.331	1.000	0.094

The restricted sample consists of participants whose performance exceeded 50% on word vs. part-word trials. The *p* value reflects a Wilcoxon test against the chance levels of 50% and of zero for accuracies and difference scores, respectively. The effect of color polarity represents a Wilcoxon test comparing all of these dependent variables as a function of color polarity. The *p* value was corrected for repeated testing using the Holm–Bonferroni method, separately for each (full or restricted) sample ($p_{\text {HB}}$). In the restricted sample, comparisons of the word vs. part-word contrast against chance are not meaningful as participants were selected based on their performance in this comparison. The effect size *r* is the rank-biserial correlation

**Fig. 3:**
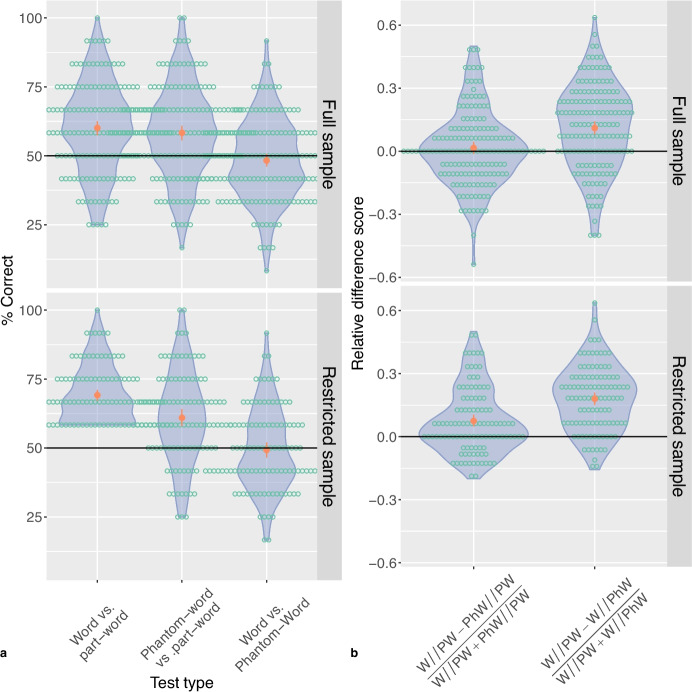
**a** Accuracy in the different trial types (words vs. part-words, phantom-words vs. part-words, and words vs. phantom-words). **b** Relative difference scores for contrasts between different trial types (word vs. part-word trials vs. phantom-word vs. part-word trials, and word vs. part-word trials vs. word vs. phantom-word trials). Both panels show the data for the full main sample (*top*) or for the restricted sample after the exclusion of participants whose performance did not exceed 50% in the word vs. part-word trials (*bottom*), collapsed across polarity contrasts (black shapes on a white background vs. white shapes on a black background). The *dots*, *error bars*, and *violin plots* represent the sample averages, 95% bootstrap confidence intervals and the distribution of the average accuracy for individual participants, respectively. *Empty circles* represent individual participants

## Results

As shown in Table 3 and Fig. 3a, participants from the main sample preferred both words and phantom-words to part-words.^1^ In contrast, they had no preference for words over phantom-words. Similar results were obtained for both color polarity types, with no discernible effect of color polarity type. These results held in both the full sample and the restricted sample. (Individual results for the different polarity types are given in Fig. S1.)

To compare performance in the different trial types, I calculated the difference scores mentioned above. As shown in Table 3 and Fig. 3b, participants from the main sample performed much better on word vs. part-word trials than on word vs. phantom-word trials, irrespective of the color polarity type. This suggests that participants find discriminations based on TPs much easier than discriminations based on frequency of occurrence, which is problematic if statistical learning leads to memory for units. (Individual results for the different polarity types are given in Fig. S2.)

However, at least in the restricted sample, performance was also somewhat better for word vs. part-word trials than for phantom-word vs. part-word trials, suggesting that one cannot rule out that participants might also have some ability to track frequencies of occurrence. However, the corresponding difference score was much smaller than that comparing words vs. part-word and word vs. phantom-word trials, and was not significant in the full sample.

As shown in Supplementary Online Material SM2, the results were similar for the student sample, except that the data was noisier.

**Table 4:** Results of generalized linear mixed models for trial-by-trial responses, for the main sample

	Log-odds			Odd ratios				
	Estimate	*SE*	*CI*	Estimate	*SE*	*CI*	*t*	*p*
**Full sample - Word//Part-Words vs. Words//Phantom-Words**								
Trial type: Words vs. Part-Words	0.530	0.092	[0.35, 0.711]	1.700	0.156	[1.42, 2.04]	5.767	< 0.001
Color polarity: white on black	0.044	0.099	[-0.151, 0.238]	1.045	0.104	[0.86, 1.27]	0.440	0.660
Trial type: Words vs. Part-Words $\times$ Color polarity: white on black	-0.093	0.131	[-0.35, 0.163]	0.911	0.119	[0.705, 1.18]	-0.713	0.476
**Full sample - Word//Part-Words vs. Phantom-Words//Part-Words**								
Trial type: Words vs. Part-Words	0.172	0.093	[-0.00962, 0.355]	1.188	0.110	[0.99, 1.43]	1.856	0.063
Color polarity: white on black	0.151	0.109	[-0.0625, 0.364]	1.162	0.126	[0.939, 1.44]	1.385	0.166
Trial type: Words vs. Part-Words $\times$ Color polarity: white on black	-0.200	0.133	[-0.46, 0.0609]	0.819	0.109	[0.631, 1.06]	-1.502	0.133
**Restricted sample - Word//Part-Words vs. Words//Phantom-Words**								
Trial type: Words vs. Part-Words	0.836	0.113	[0.616, 1.06]	2.308	0.260	[1.85, 2.88]	7.422	< 0.001
Color polarity: white on black	0.003	0.111	[-0.215, 0.221]	1.003	0.112	[0.807, 1.25]	0.028	0.978
Trial type: Words vs. Part-Words $\times$ Color polarity: white on black	-0.005	0.164	[-0.326, 0.317]	0.995	0.163	[0.722, 1.37]	-0.029	0.977
**Restricted sample - Word//Part-Words vs. Phantom-Words//Part-Words**								
Trial type: Words vs. Part-Words	0.460	0.114	[0.237, 0.683]	1.584	0.180	[1.27, 1.98]	4.047	< 0.001
Color polarity: white on black	0.209	0.117	[-0.02, 0.437]	1.232	0.144	[0.98, 1.55]	1.788	0.074
Trial type: Words vs. Part-Words $\times$ Color polarity: white on black	-0.210	0.166	[-0.536, 0.116]	0.810	0.135	[0.585, 1.12]	-1.263	0.207

Results are reported for the full sample as well as the restricted sample, where participants were excluded if their performance did not exceed 50% on the word vs. part-word trials

I confirmed these results using the generalized linear mixed models mentioned above. As shown in Table 4, the models showed that performance on word vs. part-word trials was significantly better than for word vs. phantom-word trials. They also showed that performance on word vs. part-word trials was significantly better than on phantom-word vs. part-word trials, though this predictor was significant only in the restricted sample and was only marginal in the full sample. Further, the odds ratio associated with the former contrast was almost twice as large as that from the latter contrast.

There were no main effects or interactions with polarity type. The results for the student sample were generally similar.

## Discussion

Substantial controversy revolves around the nature of the representations formed during statistical learning. On the one hand, learners might use statistical information to encode discrete and integrated units into memory. On the other hand, they might just form associations between contiguous elements, but without necessarily encoding discrete units in memory. While the evidence (at least in my view, but see e.g., Erickson et al., 2014; Graf-Estes et al., 2007; Hay et al., 2011; Isbilen et al., 2020; Karaman & Hay, 2018; Perruchet, 2019; Shoaib et al., 2018) favors the mere association view for statistical learning from sequences, there is potentially strong evidence for the memory view in the case of statistical learning from simultaneously presented visual shapes. Specifically, some studies demonstrate superior recognition of units compared to sub-units, suggesting that participants encoded the entire units.

However, and as mentioned above, the interpretation of such results is unclear, given that they are found in some experiments but not others (Fiser & Aslin, 2005; Slone & Johnson, 2018), that they might have attentional explanations (Endress, in preparation), and that a memory-less Hebbian learning model might provide an alternative interpretation (Endress & de Seyssel, under review).

To adjudicate between these competing views, I tested the predictions of the memory view and the mere-association using a paradigm that has been critical in similar discussions in the case of statistical learning of sequential regularities. Specifically, following exposure to statistically structured “scenes” composed of visual shapes, I tested recognition of words, part-words, and phantom-words. Phantom-words have the same (high) TPs as words, but, in contrast to words, never appeared during familiarization.

Participants preferred both words and phantom-words over part-words. Further, the preference for words over part-words was higher than the preference for words over phantom-words. Such results thus strongly suggest that the participants’ choices are predominantly driven by TPs rather than frequency of occurrence, and TPs are more salient that frequency of occurrence even when items are presented simultaneously. This poses a challenge for the memory view, as it suggests that participants prefer unattested items for which there is no memory representations (i.e., phantom-words) over attested items (i.e., part-words).

However, I cannot rule out some sensitivity to frequency of occurrence as well, given that the preference for words over part-words was somewhat higher than that for phantom-words over part-words. However, the odds ratio comparing word vs. part-word and word vs. phantom-word trials was twice as high than that comparing word vs. part-word and phantom-word vs. part-word trials. Further, performance was equivalent on word vs. part-word and phantom-word vs. part-word trials when all participants were included. Be that as it might, the current results suggest that participants’ choices are primarily driven by TPs rather than by frequency of occurrence.

A potential limitation of these results relates to the design of these experiments. Statistically defined units could appear in horizontal or vertical orientation. However, when the units appeared in a vertical orientation, their constituent shapes maintained their original orientation, and were not rotated together with the unit. This, in turn, might have encouraged participants to process shapes as isolated items, rather than as part of a unit. However, if learners use TPs to extract units, and if the non-rotated shapes prevented participants from recognizing the units across orientation, they should simply memorize *two* units for each word, one in a horizontal orientation, and one in a vertical orientation. As each word occurred no less than 24 times in each orientation, participants had ample opportunity to actually memorize these items. This is particularly so since, at least in language acquisition, experience is sparse. As a result, each word is exceedingly rare (e.g., Yang, 2013), and statistical learning thus must operate on sparse input. As a result, even if participants did not recognize units across orientations, the current results still show that they weight TPs higher than frequency of occurrence.

While a preference for unattested high-probability items over attested low-probability items suggest that statistical learning might not be particularly helpful for learning specific items such as words, such preferences reflect a form of generalization that might fulfill other functions. In fact, the ability to generalize has long been considered a critical ability in connectionist networks (e.g., Amit, 1989; Plunkett & Marchman, 1993; Altmann, 2002), and more recently in deep neural networks (e.g., Li, Sorscher, & Sompolinsky, 2024). Such a generalization ability might be useful for reconstructing stimuli from incomplete input (e.g., during amodal completion). It might also facilitate processing through predicting information, for example when understanding sentences (e.g., Levy, 2008; Trueswell, Sekerina, Hill, & Logrip, 1999) or more generally in cognition (Clark, 2013; Friston, 2010; Keller & Mrsic-Flogel, 2018). In fact, other authors argued that statistical learning might be particularly important for predictive processing (Sherman & Turk-Browne, 2020; Turk-Browne, Scholl, Johnson, & Chun, 2010), a function that is presumably facilitated if it is not limited to items which can be easily recognized.

Be that as it may, the current findings suggest that learners’ behavior is predominantly influenced by TPs rather than by frequency of occurrence, which limits the utility of statistical learning for remembering specific items, but might make it more useful for other purposes. It is thus urgent to directly investigate the function of statistical learning, and test its relationship with memory processing.

## Supplementary Information

Below is the link to the electronic supplementary material.

Supplementary file 1 (pdf 621 KB)

## Data Availability

Experiments, stimulus generation code, data and analysis code are available at https://github.com/aendress/phantoms_vision_simultaneous and https://figshare.com/s/559ccd8ce6bd10b24292 (DOI: 10.25383/city.26023051)

## Footnotes

1 The above chance performance in the restricted sample is meaningless, since only those participants were included who exceeded 50% on the word vs. part-word test.
