# Supplementary material for: Transitional probabilities outweigh frequency of occurrence in statistical learning of simultaneously presented visual shapes
Source: Mem Cognit. 2024 Dec 10;53(5):1497–509. doi: 10.3758/s13421-024-01665-x (PMC12307553; doi:10.3758/s13421-024-01665-x)
Supplement: Supplementary file 1 — (pdf 621 KB) [file 13421_2024_1665_MOESM1_ESM.pdf]

Supplementary Online Materials

SM1 Results separated by polarity type for the main sample

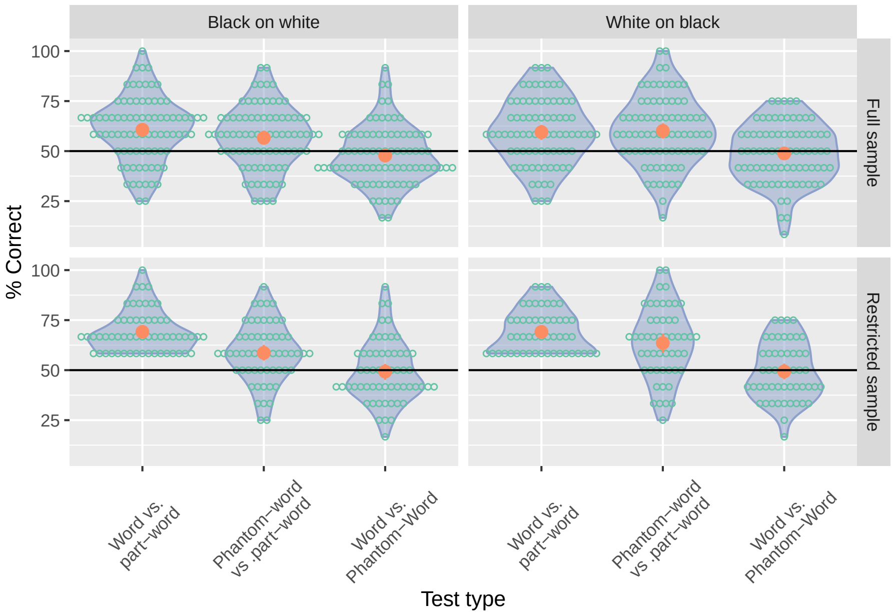

Figure S1. Accuracy in the different trial types (words vs. part-words, phantom-words vs. part-words, and words vs. phantom-words), for the full main sample (top) or after exclusion of participants whose performance did not exceed 50% in the word vs. part-word trials (bottom), for black shapes on a white background (left) and white shapes on a black background (right). The dots, error bars and violin represent the sample averages, 95% bootstrap confidence intervals and the distribution of the average accuracy for individual participants, respectively. Empty circles represent individual participants.

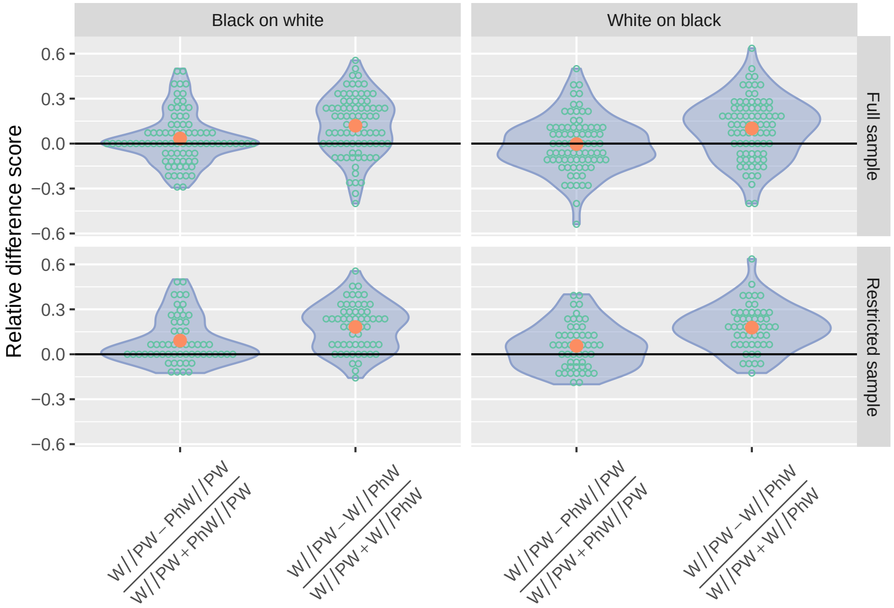

Figure S2. Relative difference scores for contrasts between different trial types (word vs. part-word trials vs. phantom-word vs. part-word trials, and word vs. part-word trials vs. word vs. phantom-word trials), for the full main sample or after exclusion of participants whose performance did not exceed 50% in the word vs. part-word trials. The dots, error bars and violon represent the sample averages, 95% bootstrap confidence intervals and the distribution of the difference scores for individual participants, respectively. Empty circles represent individual participants.

SM2 Results with the student sample

Table S1

Descriptives of accuracy scores and difference scores for the student sample. The restricted sample consists of participants whose performance exceeded 50% on word vs. part-word trials. The p value reflects a Wilcoxon test against the chance levels of 50% and of zero for accuracies and difference scores, respectively. The effect of color polarity represents a Wilcoxon test comparing all of these dependent variables as a function of color polarity. The p value was corrected for repeated testing using the Holm-Bonferroni method, separately for each (full or restricted) sample (p_{HB}). The effect size r is the rank-biserial correlation. In the restricted sample, comparisons of the word vs. part-word contrast against chance are not meaningful as participants were selected based on their performance in this comparison.

Trial type	M	SE	p_{Wilcoxon}	p_{HB}	r
**Full sample - color polarities combined (N = 50)**					
Words vs. Part-Words	56.167	2.066	0.009	0.163	0.369
Words vs. Phantom-Words	46.833	2.392	0.163	1.000	0.197
Phantom-Words vs. Part-Words	52.500	2.272	0.204	1.000	0.180
$\frac{\text{Words vs. Part-Words} - \text{Words vs. Phantom-Words}}{\text{Words vs. Part-Words} + \text{Words vs. Phantom-Words}}$	0.099	0.030	0.002	0.042	0.435
$\frac{\text{Words vs. Part-Words} - \text{Phantom-Words vs. Part-Words}}{\text{Words vs. Part-Words} + \text{Phantom-Words vs. Part-Words}}$	0.038	0.026	0.165	1.000	0.196
**Full sample - black on white (N = 23)**					
Words vs. Part-Words	57.246	2.695	0.012	0.196	0.527
Words vs. Phantom-Words	45.652	3.706	0.182	1.000	0.278
Phantom-Words vs. Part-Words	51.087	3.874	0.793	1.000	0.055
$\frac{\text{Words vs. Part-Words} - \text{Words vs. Phantom-Words}}{\text{Words vs. Part-Words} + \text{Words vs. Phantom-Words}}$	0.131	0.039	0.004	0.070	0.606
$\frac{\text{Words vs. Part-Words} - \text{Phantom-Words vs. Part-Words}}{\text{Words vs. Part-Words} + \text{Phantom-Words vs. Part-Words}}$	0.071	0.043	0.130	1.000	0.316
**Full sample - white on black (N = 27)**					
Words vs. Part-Words	55.247	3.145	0.196	1.000	0.249
Words vs. Phantom-Words	47.840	3.224	0.601	1.000	0.101
Phantom-Words vs. Part-Words	53.704	2.732	0.146	1.000	0.280
$\frac{\text{Words vs. Part-Words} - \text{Words vs. Phantom-Words}}{\text{Words vs. Part-Words} + \text{Words vs. Phantom-Words}}$	0.072	0.045	0.135	1.000	0.288
$\frac{\text{Words vs. Part-Words} - \text{Phantom-Words vs. Part-Words}}{\text{Words vs. Part-Words} + \text{Phantom-Words vs. Part-Words}}$	0.009	0.032	0.716	1.000	0.070
**Full sample - Effect of color polarity**					
Words vs. Part-Words			0.781	1.000	0.039

Table S1
(continued)

Trial type	*M*	*SE*	p_{Wilcoxon}	p_{HB}	*r*
Words vs. Phantom-Words			0.553	1.000	0.084
Phantom-Words vs. Part-Words			0.392	1.000	0.121
$\frac{\text{Words vs. Part-Words} - \text{Words vs. Phantom-Words}}{\text{Words vs. Part-Words} + \text{Words vs. Phantom-Words}}$			0.329	1.000	0.138
$\frac{\text{Words vs. Part-Words} - \text{Phantom-Words vs. Part-Words}}{\text{Words vs. Part-Words} + \text{Phantom-Words vs. Part-Words}}$			0.250	1.000	0.163
**Restricted sample - color polarities combined (N = 27)**					
Words vs. Part-Words	66.975	1.601	NA	NA	NA
Words vs. Phantom-Words	51.543	3.683	0.602	1.000	0.100
Phantom-Words vs. Part-Words	54.938	2.410	0.019	0.203	0.452
$\frac{\text{Words vs. Part-Words} - \text{Words vs. Phantom-Words}}{\text{Words vs. Part-Words} + \text{Words vs. Phantom-Words}}$	0.154	0.038	0.001	0.014	0.647
$\frac{\text{Words vs. Part-Words} - \text{Phantom-Words vs. Part-Words}}{\text{Words vs. Part-Words} + \text{Phantom-Words vs. Part-Words}}$	0.106	0.028	0.001	0.016	0.638
**Restricted sample - black on white (N = 12)**					
Words vs. Part-Words	67.361	2.262	NA	NA	NA
Words vs. Phantom-Words	52.778	5.392	0.623	1.000	0.142
Phantom-Words vs. Part-Words	47.917	4.167	0.765	1.000	0.086
$\frac{\text{Words vs. Part-Words} - \text{Words vs. Phantom-Words}}{\text{Words vs. Part-Words} + \text{Words vs. Phantom-Words}}$	0.141	0.051	0.019	0.203	0.679
$\frac{\text{Words vs. Part-Words} - \text{Phantom-Words vs. Part-Words}}{\text{Words vs. Part-Words} + \text{Phantom-Words vs. Part-Words}}$	0.180	0.045	0.004	0.051	0.839
**Restricted sample - white on black (N = 15)**					
Words vs. Part-Words	66.667	2.381	NA	NA	NA
Words vs. Phantom-Words	50.556	5.355	0.875	1.000	0.041
Phantom-Words vs. Part-Words	60.556	1.968	0.002	0.032	0.797
$\frac{\text{Words vs. Part-Words} - \text{Words vs. Phantom-Words}}{\text{Words vs. Part-Words} + \text{Words vs. Phantom-Words}}$	0.165	0.058	0.018	0.203	0.608
$\frac{\text{Words vs. Part-Words} - \text{Phantom-Words vs. Part-Words}}{\text{Words vs. Part-Words} + \text{Phantom-Words vs. Part-Words}}$	0.047	0.029	0.143	1.000	0.378
**Restricted sample - Effect of color polarity**					
Words vs. Part-Words			0.698	1.000	0.075
Words vs. Phantom-Words			0.825	1.000	0.043
Phantom-Words vs. Part-Words			0.008	0.107	0.508
$\frac{\text{Words vs. Part-Words} - \text{Words vs. Phantom-Words}}{\text{Words vs. Part-Words} + \text{Words vs. Phantom-Words}}$			0.807	1.000	0.047
$\frac{\text{Words vs. Part-Words} - \text{Phantom-Words vs. Part-Words}}{\text{Words vs. Part-Words} + \text{Phantom-Words vs. Part-Words}}$			0.011	0.130	0.491

The results for the student sample depended somewhat on whether the full sample or the restricted sample were analyzed, and on whether a correction for repeated testing was applied. This is presumably due to a combination of the limited sample size, and the high proportion of participants paying no attention to the stimuli.

The results for raw accuracy scores are given in Table S1 and Figure S3a. (Individual results for the different polarity types are given in Figure S4.)

While participants in the restricted sample preferred words over part-words (unsurprisingly, given that only those participants were included who exceeded 50% on the word vs. part-word test), this preference was only significant in the full sample when the Holm-Bonferroni correction was not applied. In the restricted sample, participants also preferred phantom-words to part-words, though this preference survived the Holm-Bonferroni correction only when white shapes were presented on a black background. In the full sample, this preference was not significant. Participants had no preference for words over phantom-words. There was no discernible effect of color polarity type.

To compare performance in the different trial types, I calculated the difference scores mentioned above. As shown in Table S1 and Figure S3b, participants from the student sample performed much better on word vs. part-word trials than on word vs. phantom-word trials. While this effect was generally significant before applying the Holm-Bonferroni correction, it did not survive this correction for all polarity types. Be that as it may, these results suggest that participants find discriminations based on TPs much easier than discriminations based on frequency of occurrence, which is problematic if statistical learning leads to memory for units. (Individual results for the different polarity types are given in S5.)

However, at least in the restricted sample, performance was also somewhat better for word vs. part-word trials than for phantom-word vs. part-word trials, suggesting that I cannot rule out that participants might also have some ability to track frequencies of occurrence. However, the corresponding difference score was much smaller than that comparing words vs. part-word and word vs. phantom-word trials, and was not significant in the full sample.

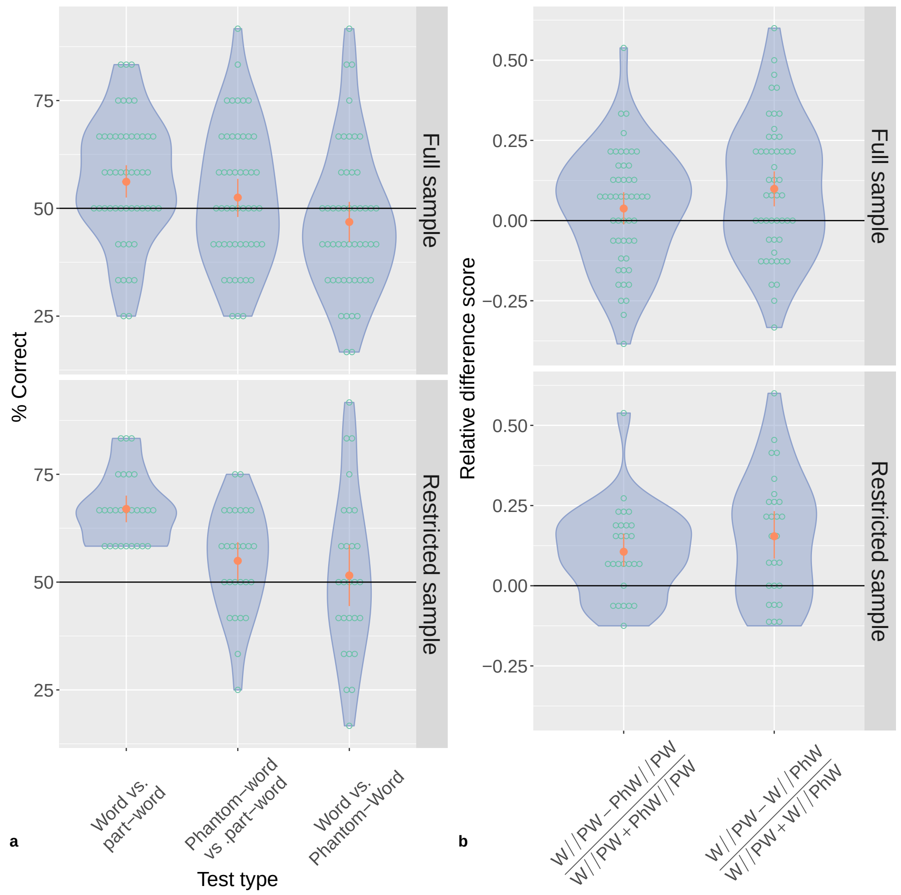

Figure S3. (a) Accuracy in the different trial types (words vs. part-words, phantom-words vs. part-words, and words vs. phantom-words), (b) Relative difference scores for contrasts between different trial types (word vs. part-word trials vs. phantom-word vs. part-word trials, and word vs. part-word trials vs. word vs. phantom-word trials). Both panels show the data for the full student sample (top) or after exclusion of participants whose performance did not exceed 50% in the word vs. part-word trials (bottom), collapsed across polarity contrasts (black shapes on a white background vs. white shapes on a black background). The dots, error bars and violon represent the sample averages, 95% bootstrap confidence intervals and the distribution of the average accuracy for individual participants, respectively. Empty circles represent individual participants.

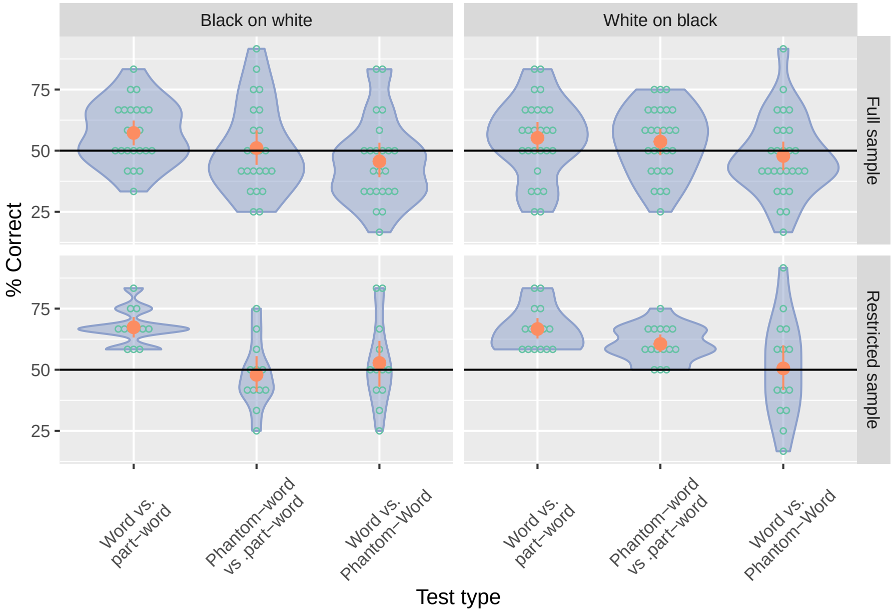

Figure S4. Accuracy in the different trial types (words vs. part-words, phantom-words vs. part-words, and words vs. phantom-words), for the full student sample (top) or after exclusion of participants whose performance did not exceed 50% in the word vs. part-word trials (bottom), for black shapes on a white background (left) and white shapes on a black background (right). The dots, error bars and violon represent the sample averages, 95% bootstrap confidence intervals and the distribution of the average accuracy for individual participants, respectively. Empty circles represent individual participants.

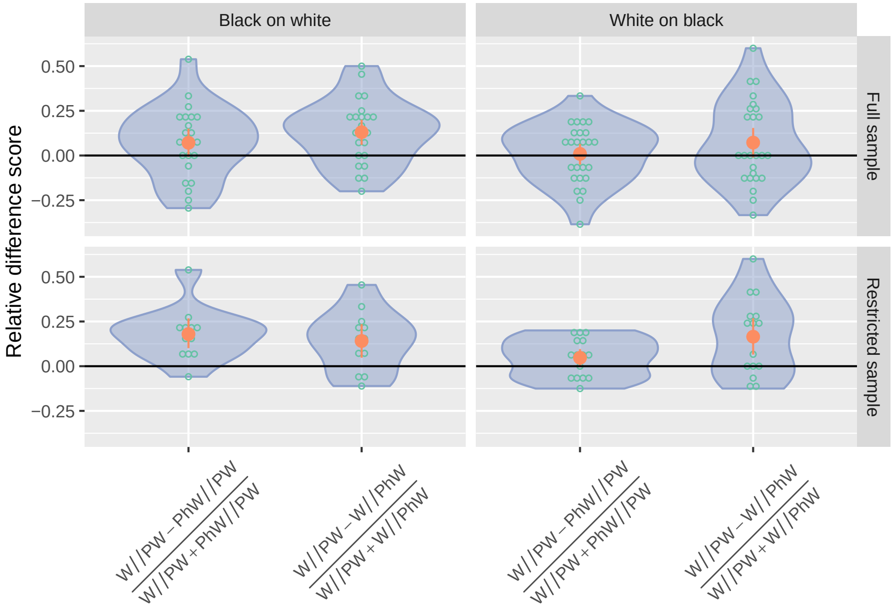

Figure S5. Relative difference scores for contrasts between different trial types (word vs. part-word trials vs. phantom-word vs. part-word trials, and word vs. part-word trials vs. word vs. phantom-word trials), for the full student sample or after exclusion of participants whose performance did not exceed 50% in the word vs. part-word trials. The dots, error bars and violon represent the sample averages, 95% bootstrap confidence intervals and the distribution of the difference scores for individual participants, respectively. Empty circles represent individual participants.

Table S2

Results of generalized linear mixed models for trial-by-trial responses, for the student sample. Results are reported for the full sample as well as the restricted sample, where participants were excluded if their performance did not exceed 50% on the word vs. part-word trials.

	Log-odds			Odd ratios				
	Estimate	*SE*	*CI*	Estimate	*SE*	*CI*	*t*	*p*
**Full sample - Word//Part-Words vs. Words//Phantom-Words**								
Trial type: Words vs. Part-Words	0.475	0.173	[0.136, 0.814]	1.608	0.278	[1.15, 2.26]	2.743	0.006
Color polarity: white on black	0.090	0.183	[-0.269, 0.448]	1.094	0.200	[0.764, 1.57]	0.489	0.624
Trial type: Words vs. Part-Words × Color polarity: white on black	-0.172	0.235	[-0.633, 0.288]	0.842	0.198	[0.531, 1.33]	-0.733	0.463
**Full sample - Word//Part-Words vs. Phantom-Words//Part-Words**								
Trial type: Words vs. Part-Words	0.251	0.172	[-0.0862, 0.589]	1.286	0.221	[0.917, 1.8]	1.460	0.144
Color polarity: white on black	0.106	0.176	[-0.239, 0.452]	1.112	0.196	[0.787, 1.57]	0.602	0.547
Trial type: Words vs. Part-Words × Color polarity: white on black	-0.188	0.234	[-0.647, 0.271]	0.828	0.194	[0.523, 1.31]	-0.804	0.421
**Restricted sample - Word//Part-Words vs. Words//Phantom-Words**								
Trial type: Words vs. Part-Words	0.614	0.244	[0.136, 1.09]	1.848	0.451	[1.15, 2.98]	2.516	0.012
Color polarity: white on black	-0.089	0.226	[-0.532, 0.354]	0.915	0.207	[0.587, 1.42]	-0.394	0.693
Trial type: Words vs. Part-Words × Color polarity: white on black	0.058	0.327	[-0.583, 0.698]	1.059	0.346	[0.558, 2.01]	0.176	0.860
**Restricted sample - Word//Part-Words vs. Phantom-Words//Part-Words**								
Trial type: Words vs. Part-Words	0.808	0.244	[0.33, 1.29]	2.243	0.547	[1.39, 3.62]	3.315	0.001
Color polarity: white on black	0.512	0.226	[0.0691, 0.955]	1.669	0.377	[1.07, 2.6]	2.266	0.023
Trial type: Words vs. Part-Words × Color polarity: white on black	-0.543	0.328	[-1.19, 0.0997]	0.581	0.191	[0.305, 1.1]	-1.656	0.098

I confirmed these results using the generalized linear mixed models above. As shown in Table S2, the models showed that performance on word vs. part-word trials was significantly better than for word vs. phantom-word trials. They also showed that performance on word vs. part-word trials was significantly better than on phantom-word vs. part-word trials, though this predictor was significant only in the restricted sample but not for the full sample. In the model comparing word vs. phantom-word trials and word vs. part-words and phantom-word vs. part-word trials, performance was somewhat better when white shapes were presented on a black background. There were no other main effects or interactions with polarity type.
